# Effects of Sanctions on Divorce in Iran: A Social Challenge

**DOI:** 10.34172/aim.35251

**Published:** 2026-02-01

**Authors:** Habibollah Azarbakhsh, Fatemeh Rezaei, Andishe Hamedi

**Affiliations:** ^1^Department of Community Medicine, School of Medicine, Ahvaz Jundishapur University of Medical Sciences, Ahvaz, Iran; ^2^Research Center for Social Determinants of Health, Jahrom University of Medical Sciences, Jahrom, Iran; ^3^Student Research Committee, Shiraz University of Medical Sciences, Shiraz, Iran

## Dear Editor

 Economic sanctions and the resulting financial pressures, including rising inflation, unemployment, high housing and wedding costs, have placed significant pressure on young people and families. This economic pressure has led to family disputes and an increase in divorce rates.^1^

 Divorce rates are on the rise globally, with industrialized countries having higher divorce rates than developing countries. According to statistics, the average crude divorce rate in the world in 2021 was 1.8.^2^ However, divorce rates vary widely across countries, and these differences must be considered within the context of the specific cultural and historical factors of each society.^3^

 In Iran, we have also witnessed an increase in divorce rates over the past decade, with this trend evident in all provinces of the country.^4^ The divorce rate has increased from 8 cases per 100 marriages in 1996^5^ to 28 cases in 2016.^6^ Also, by the end of 2016, the crude divorce rate increased to 2.27 per thousand people,^7^ and according to the latest statistics from the Statistical Center of Iran in 2019, the divorce rate is about 32.9%, with one divorce recorded for every four marriages.^8^ This increase has coincided with recent severe economic and social pressures in the country.^8^

 In addition to the social effects, divorce has a wide range of effects on the physical and mental health of parents and their children. Divorced individuals are more likely to experience depression, anxiety, aggression, social isolation, and other psychological problems.^9^ Children of divorced families also face a higher risk of behavioral disorders, academic problems, addiction, and problems in adult relationships.^10^

 To address this complex social challenge, it is recommended to:

1. Implement economic support policies such as unemployment insurance and inflation control to reduce financial pressure on families and consequently reduce divorce;
2. Improve economic conditions and create sustainable job opportunities, especially for the youth and women;
3. Reduce the costs of infertility treatment and provide effective financial and social support to families;
4. Promote a culture of family preservation and increase public awareness about the negative consequences of divorce;
5. Facilitate marriage and lower the age of marriage by providing incentives and planned support;
6. Provide psychological and educational services to couples to cope with financial stress and maintain the mental health of the family.

Implementing these strategies can prevent the excessive growth of the divorce rate and maintain the strength of the family foundation, especially in conditions of economic pressure resulting from widespread sanctions.
